# Daytime Paddock Behaviour of Alpacas Raised in an Australian Extensive Production System: A Pilot Study

**DOI:** 10.3390/ani15162357

**Published:** 2025-08-11

**Authors:** Imogen Boughey, Evelyn Hall, Russell Bush

**Affiliations:** Sydney School of Veterinary Science, The University of Sydney, 425 Werombi Road, Camden, NSW 2567, Australia

**Keywords:** alpaca, camelid, grazing system, paddock behaviour

## Abstract

The Australian alpaca industry is continuing to develop as an alternative fibre industry to the traditional merino or angora industries. This study aimed to investigate herd behaviour in an extensive system in south eastern Australia. Healthy adult female alpacas (Huacaya n = 32, Suri n = 32) over two years old were inducted into the trial and kept together across a 10 month period. A total of 5 animals were removed during the study due to illthrift or death unrelated to the study. GoPro cameras were set up at 5 locations in the paddock for 3 days in the middle of every season (Summer, Autumn, Winter, Spring) to record alpaca behaviour without a human observer present. Visual observations were taken at 0800, 1000, 1100, 1300, 1500 for 60 min. Behaviour observations were taken every 5 min from the videos according to a prepared ethogram. A count of animals exhibiting each behaviour was recorded at each time point within each of the designated 60-minute periods. A generalised linear mixed-effects model (GLMM) was run on binary data for each behaviour. Behaviours that returned a predicted proportion of over 0.10 for all seasons were used in an ordinal logistic regression that was then utilised to determine the effect of the season, time of day, and weather conditions on the number of animals. Season significantly impacted the number of alpacas grazing, resting, and standing (*p* < 0.0001). Alpacas were more likely to be grazing throughout the day in cooler seasons (autumn, winter) and resting in the warmer parts of the day in summer and spring. The time of day impacted the proportion of alpacas resting and grazing (*p* < 0.05) but not standing (*p* = 0.4432). This study highlights that alpacas spend the majority of the daylight hours grazing, with some variability across different seasons, which may impact ideal management practices to optimise production in an extensive system.

## Simple Summary

The alpaca industry in Australia began in the 1980s and 1990s and continues developing as an alternative natural fibre industry. This study aimed to create a baseline for alpaca herd behaviour in an extensive production system in southeastern Australia. Sixty-four adult female alpacas, 32 Huacaya and 32 Suri, were inducted into the trial and kept together across 10 months. Visual monitoring occurred using GoPro cameras for 3 days in the middle of every season in the same paddock to record paddock behaviour without a human presence. Videos were taken five times per day at 0800, 1000, 1100, 1300, 1500 for 60 min with behaviour observations recorded every 5 min. Grazing, resting and standing were the most common behaviours. Alpacas were more likely to be seen grazing at any time throughout the day in the cooler seasons (autumn and winter) and resting in the warmer parts of the day during summer and spring. The time of day impacted the number of alpacas seen resting or grazing at any point in time, but not the number of alpacas standing still. This study has outlined the common paddock behaviours for alpacas in southeastern Australia, highlighting that alpacas spend the majority of daylight grazing compared to other observed behaviour.

## 1. Introduction

Alpacas (*Vicugna pacos*) are New World Camelids that are the domesticated descendants of the vicugna and are closely related to llamas and the guanaco [1]. Alpacas originate from South America in high-altitude regions, where they are used for both meat and fibre production [2]. The alpaca industry began to develop in Australia from the 1980s as an alternative fibre industry and continues to grow in size, with an estimated 350,000 alpacas currently in Australia [3,4]. The Australian alpaca population is mainly comprised of small- (≥250 alpacas) to medium-scale (50–249 alpacas) properties with an increasing number of larger-sized enterprises (≥250 alpacas) operating as an extensive production system [5].

Understanding the grazing patterns and pasture utilisation of sheep and cattle in pasture-based systems has enabled the development of long-term sustainable management strategies to improve animal productivity and land management [6,7]. Developing a great understanding of normal animal behaviour enables us to recognise behaviours that deviate from normal and may indicate early signs of illness [8,9]. Early recognition of illness facilitates early intervention and improved health outcomes, as well as mitigating the spread of disease [8,9]. Improving the knowledge of sheep and cattle behaviour regarding handling has led to the development of low-stress handling techniques and systems and infrastructure designed to minimise stress [8,10]. Reducing stress associated with handling and husbandry practices improves animal welfare and production outcomes, as animals that experience lower stress levels are more likely to go back to feed faster, negating negative impacts on production [8,10]. A large portion of the existing literature on the grazing behaviour of alpacas is focused on feed selection in South American environments [11,12,13]. Identifying that alpacas have the ability to consume and thrive on a higher level of low-quality forage compared with other small ruminants in their native environments has provided valuable insight into managing alpacas when low-quality feed is available. However, this information is specific to South American production systems [11,13], highlighting the need for further research in other production systems to improve management recommendations.

Across the world, it is common for alpacas to co-inhabit with sheep, as well as llamas [11,14,15]. The ability of alpacas to successfully co-inhabit and guard other grazing species, such as sheep, has led to their increased use in Australia [16]. Early research during the development of the alpaca industry was focused on cohabitation studies with sheep [15]. When alpacas co-inhabit with sheep, they spend most of their time grazing (57%), with less time spent on other activities such as resting (27%) [17]. Zabek et al. [18] also reported that alpacas with cria at foot displayed grazing as the dominant behaviour. These results, despite different production conditions (herd guardians, smallholder farms, and being housed inside at night), highlight that grazing behaviours occur more frequently than other behaviours, including resting and standing.

However, there is limited information on the grazing and paddock behaviour of alpacas in an extensively raised system, especially in Australia. Current research has focused primarily on alpaca behaviour with regards to sheep, either in a co-grazing scenario [15,17] or as guardian animals [16,19,20]. As the Australian alpaca industry continues to develop with an increase in larger-scale properties [5], it is important to understand baseline herd behaviour in an extensive production system of both alpaca breeds to improve management recommendations and producer education resources. This study aimed to provide a baseline of herd paddock behaviours of alpacas raised in an extensive production system in Southeastern Australia.

## 2. Materials and Methods

### 2.1. Animal Usage and Location

A total of 64 adult female alpacas (32 Suri and 32 Huacaya) were inducted into the trial conducted at a property located in the Southern Highlands, New South Wales, Australia. At the time of induction, animals were over 2 years old and had a body condition score (BCS) greater than 2 (scale 1–5). The study was conducted across a 10-month period from January to October 2024 to collect data across all seasons. A total of 5 animals were removed from the study at different time points due to ill-thrift (2) or death due to causes unrelated to the study (3). The animals used in this study experienced regular interactions with humans and had two (2) days of acclimatisation to the presence of the cameras in a smaller paddock prior to starting the behaviour monitoring. The alpacas remained together for the duration of the study. The University of Sydney Animal Ethics Committee (ethics number 2023/2333) granted animal ethics approval. The weather conditions were recorded at each time point based on visual observations in the video recordings. The weather was classified as overcast, raining, or sunny.

### 2.2. Behaviour Monitoring

**Figure 1:**
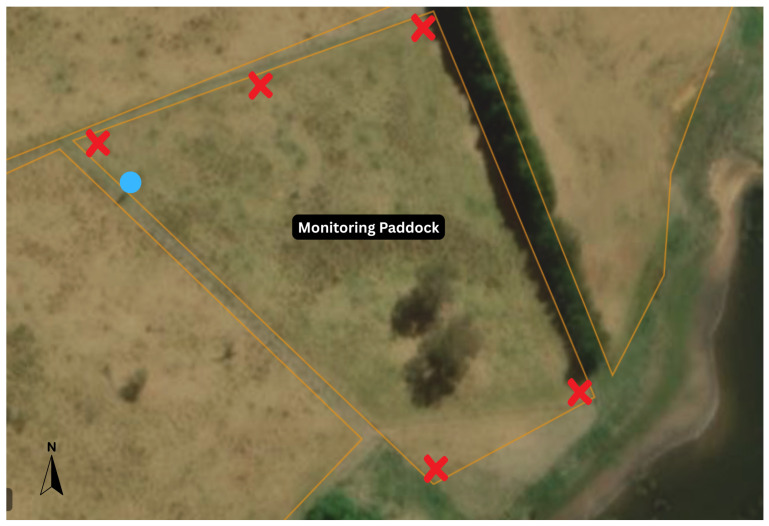
Locations of cameras and water sources in the monitoring paddock. A red X indicates approximate camera locations, and the blue circle indicates the approximate water trough location.

Visual behaviour measurements were taken between 0800 and 1500 AEST during the middle of each season for 3 days. The paddock used for the behaviour monitoring was 1.01 ha. Natural alpaca behaviour without the impact of human presence was recorded through 5 wide-lens cameras placed at each corner of the paddock and an additional camera at a high vantage point (Figure 1). In summer, 2 GoPro Hero 11 Black (GoPro Inc., San Mateo, CA, USA) and 3 GoPro Hero 4 (GoPro Inc.) cameras were used. Malfunctions with the GoPro Hero 4 cameras due to overheating resulted in some missing data due to date-limited usable footage. These cameras were replaced with 3 GoPro Hero 12 Black cameras for the remainder of the study. Video collection occurred in the middle of each season. Recordings ran for 60 min across 5 time periods, starting at 0700, 0900, 1100, 1300, and 1500 AEST in the middle of each season. In summer, the GoPro Hero 4 cameras were manually started, resulting in the first and last 5 min measurements being removed due to human presence for some recordings. In all other seasons, GoPro Hero 11 and 12 cameras were used with preset start times and duration limits, allowing the collection of SD cards and the reset of cameras between monitoring periods without influencing alpaca behaviour during monitoring.

Behaviour observations were taken every 5 min from the videos, checking all camera footage. Observations were conducted by a trained observer familiar with presentations of alpaca behaviour on a prepared ethogram (Table 1). The behavioural states included in this study were developed based on the authors’ extensive experience working within the Australian alpaca industry. Behaviour states were defined as behaviours occurring for more than one minute to ensure that clear identification was impossible for moving behavioural states [21]. The following behaviours were included in the ethogram: cush (a seated position where the legs are folded under the body that is specific to the alpaca), lying down, standing still, walking, grazing, chewing cud while standing, chewing cud while in cush, drinking, rolling, urinating or defecating, scratching, other (with description), and out of sight (Table 1). A count of animals exhibiting each behaviour was recorded at each time point within each of the designated 60 min periods. Any animals that could not be seen or were displaying behaviour that was not distinguishable were recorded as out of sight. For further analysis, cush, lying down, chewing cud in cush, and chewing cud while standing were all grouped together to be analysed as resting behaviours.

### 2.3. Statistical Analysis

Statistical analyses were conducted in R (Version 4.4.2 [22]) and Excel (Version 16.98 [23]). A generalised linear mixed-effects model (GLMM) was run on binary data for each behaviour using the lm4 package [24]. A GLMM was run to assess the effect of the season on the binary (yes/no observed) data obtained for each behaviour to generate predicted proportions for each behaviour at each season (summer/autumn/winter/spring). Behaviours that returned a predicted proportion of over 0.10 for all seasons were considered common and were used for further analysis. The data for the selected common behaviours (resting, grazing, and standing still) were categorised into groups that indicated the percentage of the herd observed performing a behaviour. The categories were 0%, ≤25%, 26–50%, 51–75%, and 75–100%. Ordinal logistic regression was then utilised to determine the effect of the season, time of day, and weather conditions on the number of animals exhibiting the behaviours using the ordinal package in R [25]. A *p*-value of >0.05 was considered significant.

## 3. Results

Walking, drinking, rolling, urinating, defecating, and scratching behaviours returned a predicted proportion under 0.10 and were not included in further analysis. Resting (including cush, lying down, chewing cud in cush, and chewing cud while standing), grazing, and standing still returned predicted proportions greater than 0.10 and were used in further analysis.

### 3.1. Resting Behaviours

**Figure 2:**
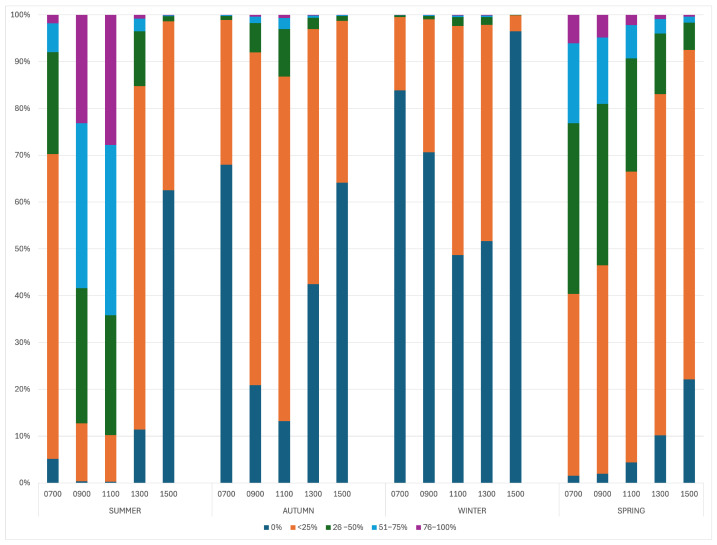
The effect of the time of day and season on the percentage of the herd exhibiting resting behaviours.

There was a significant impact of the season on the number of animals displaying resting behaviours (*p* < 0.001) (Table 2). Alpacas were more likely to spend more time resting in the middle of the day during summer and spring compared to winter and autumn (Figure 2). Alpacas were 2.07 times (95% CI 1.43–2.97) more likely to exhibit resting behaviours in rain compared to overcast conditions, and they were 5.04 (95% CI 3.95–6.42) times more likely to show resting behaviours in sunshine compared to overcast conditions. Odds ratios and *p*-values can be seen in Table 2.

### 3.2. Grazing

**Figure 3:**
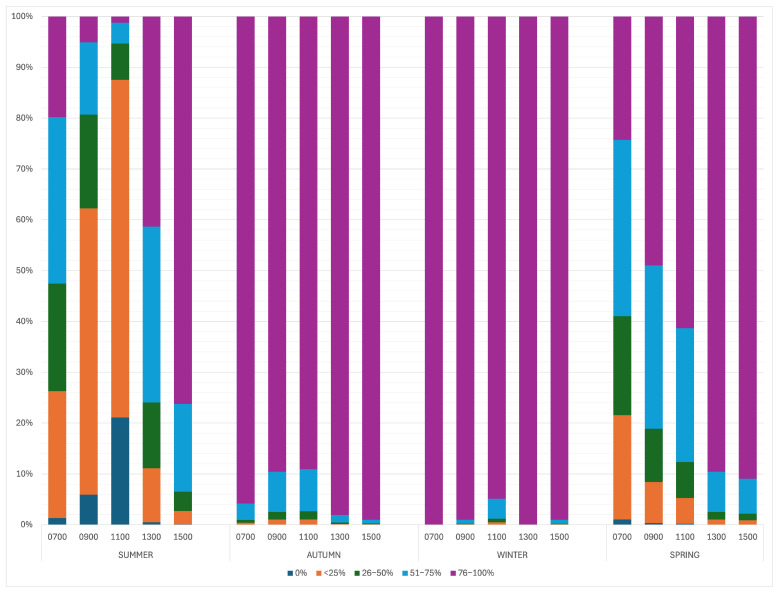
The effect of the time of day and season on the percentage of the herd grazing.

The season significantly impacted the number of alpacas grazing (*p* < 0.0001), with a higher proportion of the herd being more likely to be grazing at any time point during autumn and winter (Figure 3). The time of day had a significant impact on the proportion of alpacas grazing (*p* < 0.0001). Alpacas were 3.48 (95% CI 2.43–5.18) times more likely to be grazing at 1300 than at 0700 and 5.73 times more likely at 1500 (95% CI 3.86–8.92). They were more likely to graze later (1300–1600) in the day (Table 2). Weather significantly affected the proportion of alpacas grazing (*p* < 0.001). Alpacas were 0.55 times (95% CI 0.34–0.89) more likely to be grazing in rain compared to overcast conditions and 0.19 (95% CI 0.13–0.26) times more likely to graze in sunshine compared to overcast conditions. Odds ratios and *p*-values can be seen in Table 2.

### 3.3. Standing Still

**Figure 4:**
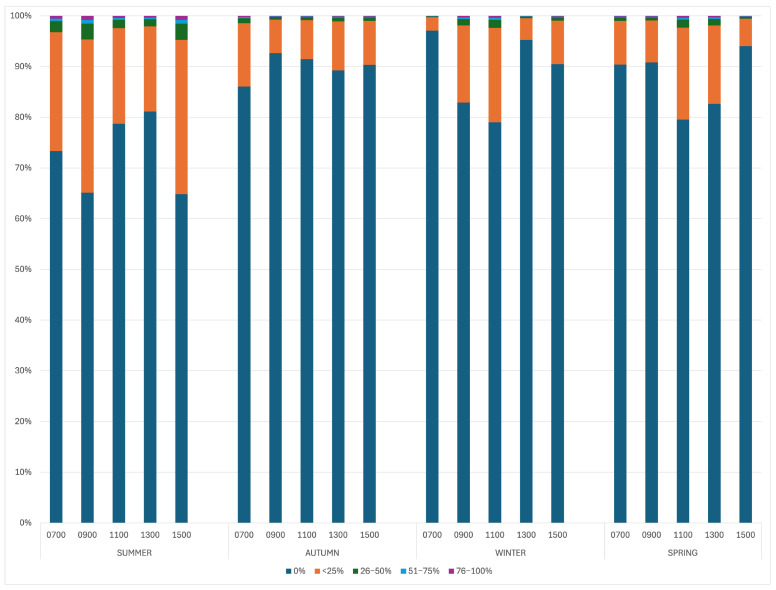
The effect of the time of day and season on the percentage of the herd standing still.

The proportion of alpacas standing was impacted by the season (*p* < 0.0001), with more animals standing still in the warmer seasons compared to winter and autumn (Figure 4). The number of alpacas standing still was not impacted by the time of day (*p* = 0.4432) or weather conditions (*p* = 0.4354) (Table 2).

## 4. Discussion

Alpaca paddock behaviour at an independent herd level has not previously been researched in Australia. Prior research has focused on alpaca behaviour when used as herd guardians for sheep [17], whereas this pilot study investigated alpaca behaviour when grazed on their own in an extensive production system. In another first, this study utilised GoPro cameras to record alpacas on video for 60 min periods during daylight hours to assess their behaviour without human presence. This provided an opportunity to assess the feasibility of using this technology for behaviour monitoring in an extensive system, particularly where access to power is limited. From this, it was found that grazing is the dominant behaviour of alpacas during daylight hours, with resting and standing also being common. Grazing is also reported to be one of the dominant daytime behaviours observed in sheep, but sheep conduct grazing consistently over a 24 h period with more discrete meal times compared to alpacas, highlighting the differences between the two species, which are commonly grazed together [16,26]. Anecdotal evidence from alpaca producers suggests that their understanding of behaviour and appropriate management practices are often based on sheep, as they are a similar small ruminant species.

### 4.1. Grazing Behaviour

This study found that alpacas spent more time grazing compared to resting or standing during the day. Alpacas do the majority of their grazing during daylight, which differs slightly from the behavior of sheep, as sheep spread their grazing over a longer time frame, including at night [7,27]. Sheep have been reported to have distinct grazing peaks at sunrise and sunset, whilst alpacas are observed to be resting or ruminating in these timeframes [26,27].

In Poland and New Zealand, alpacas were found to do most of their grazing during daylight hours (0700–1850) [26,28]. In a study by Kapustka and Budzynska [28], alpacas were housed in stables overnight, which is a key difference in management practices compared to this study. However, in a New Zealand study by Sharp et al. [26], alpacas were observed in a paddock for 24 h periods, the authors reported a similar trend with minimal grazing occurring after sunset. It is expected that there will be minimal to no grazing at night in Australian alpacas in a similar extensive production system. However, this would require further research to confirm this herd behaviour under Australian conditions.

Understanding grazing behaviour and key grazing time periods can assist in improving production and maintaining animal health. This especially applies during periods where adjustments to uninterrupted grazing occur, such as during dry periods when pasture is limited and a supplement is supplied [29]. Developing supplement feeding regimes, including those considering the feed type and timing to cause minimal disruption to the animals’ digestive system, is important for maintaining health [29,30]. In alpacas, strategically aligning feeding times with natural peaks in grazing activity will likely best fit with alpaca behavioural routines and may also minimise supplement waste, as the feed is supplied at a time when alpacas would naturally prefer to eat.

Other factors shown to influence grazing behaviour in other grazing livestock, including sheep, include the amount and location of shade and water troughs [6]. However, there is no research on this in alpacas in Australia. This study found that alpacas were more likely to be resting in sunny conditions than overcast or rainy conditions; however, the amount of time spent in the shade or sun was not recorded. As this study collected baseline herd behaviour across the four seasons, all observations were collected in the same paddock. Hence, factors such as varying water locations and shade locations were not investigated. It is also worth noting that time spent drinking was not regularly recorded on the cameras in this study. The impact of paddock layout, including differing shade and water locations, is an area for future research on alpaca behaviour to optimise production and animal welfare.

### 4.2. Effect of the Season on Alpaca Herd Behaviour

In this study, alpacas were more likely to be grazing in the afternoon between 1300 and 1550 than early in the morning. In the warmer seasons (summer and spring), alpacas were more likely to graze in the late afternoon compared to the morning and the middle of the day, which is similar to what happens in New Zealand [26]. Alpacas in Australia are likely to graze more frequently and for longer periods when the conditions are cooler, such as in autumn and winter. The season had a significant effect (*p* <0.001) for all behaviours, and this is likely due to changes in daily temperatures, as well as the length of daylight. As alpacas graze predominantly during daylight [26,28] and are more likely to graze in rainy than in sunny conditions, it is plausible that grazing occurred more often in the winter and autumn monitoring periods due to the more favourable conditions (lower daily temperatures), as well as shorter days, leading to a more condensed grazing period, which was able to be captured on camera. Although resting behaviours were commonly observed in this study, the proportion of alpacas resting at any one time was lower than that of those engaging in grazing behaviours, except in Summer, which has also been observed in sheep [31] and is likely due to the warmer weather and increased shade-seeking behaviours.

### 4.3. Resting and Standing Behaviours

Resting behaviour in this study included ruminating (chewing cud), cush, and standing, as well as lying down and sitting in cush, as the alpacas were at rest and not in an alert state. Standing was treated as a separate behaviour, as animals were still in an alert state. Alpacas in this study were about twice as likely to be resting at 0900 and 1100 compared to 0700, and they were one-third as likely to be resting in the afternoon (1500) compared to the morning (0700). This differs from New Zealand, where alpacas spent significantly more time ruminating early in the morning (0100–0650) [26]. Sharp et al. [26] found that alpacas and sheep grazed together in New Zealand spent more time ruminating and standing compared to grazing [7], which was not seen in this study.

Weather conditions impacted resting behaviours, with alpacas being more likely to be resting when conditions were sunny compared to being overcast. These effects have also been reported in sheep, with routine grazing and ruminating behavioural trends being impacted by changes in weather and climatic conditions [7]. The impact of weather conditions on alpacas raised in Australia would benefit from further research across different climate regions to assess the degree of the impact of weather on herd behaviour.

### 4.4. Technology Use and Limitations

Cameras used in this study were unable to record behaviours during the night. In addition, older GoPro models overheated in the summer period, resulting in some footage not being recorded. Replacing these with newer cameras ensured footage capture in subsequent seasons, but night vision was not possible. Future research would benefit from trialling additional technology, such as GPS tracking technology, to monitor alpaca behaviour on farms. Although there were challenges with camera use, there were clear benefits in the ability to observe alpaca behaviour in an extensive paddock environment without constant human presence and limited human interaction. This provided a cohesive account of natural herd behaviours in an extensive production system.

## 5. Conclusions

Alpacas raised in an extensive production system in Australia exhibit resting, grazing, and standing as the most frequently observed behaviours. Alpacas are more likely to spend time grazing in winter and autumn compared to summer, showing a preference for grazing for longer periods in cooler conditions. They are also more likely to graze in the afternoon (between 1300 and 1600 AEST) compared to the morning, when they are most likely resting (between 0900–1200 AEST). Understanding the grazing behaviour of Australian-raised alpacas enables the future development of suitable management practices, such as supplement feeding times, to optimise health and productivity.

## Figures and Tables

**Table 1:** Definition of behavioural states used in behaviour observations of alpacas raised in an extensive production system in Australia.

Behaviour	Description
Chewing Cud in Cush	The alpaca is seated in cush, and a chewing motion can be seen without the presence of grazing.
Chewing Cud Standing	The alpaca is standing, and a chewing motion can be seen without the presence of grazing.
Cush	A seated position where the legs are folded under the body and the alpaca’s body remains upright. The head/neck may be up or down.
Lying Down	The alpaca is lying on its side with legs out (not under its body). The head/neck is commonly on the ground but may be lifted.
Standing Still	The alpaca is standing still (not chewing cud).
Grazing	The alpaca is actively grazing (may include slowly walking while the head is down). The alpaca can be seen taking “bites” of the pasture or feed.
Walking	The alpaca is walking without grazing.
Drinking	The alpaca is drinking (from a trough or water source).
Rolling	The alpaca is seen actively moving on its back, generally side to side, commonly in dirt or short grass.
Urinating or Defecating	The alpaca is seen urinating or defecating.
Scratching	The alpaca is using either its back legs or teeth to scratch another area of its body.

**Table 2:** The effects of the season, time, and weather on resting, grazing, and standing behaviours.

			Odds Ratio	LCI	UCI	*p*-Value
	Season	WINTER	-	-	-	
		AUTUMN	0.26	0.11	0.62	
		SPRING	0.03	0.01	0.06	<0.001
		SUMMER	0.01	0.00	0.02	
	Time	0700	-	-	-	
		0900	2.00	1.43	2.81	
Resting		1100	2.10	1.51	2.93	<0.001
		1300	0.93	0.68	1.27	
		1500	0.31	0.22	0.42	
	Weather	Overcast	-	-	-	
		Rain	2.07	1.43	2.97	<0.001
		Sun	5.04	3.96	6.42	
	Season	WINTER	-	-	-	
		AUTUMN	0.26	0.11	0.62	
		SPRING	0.03	0.01	0.06	<0.0001
		SUMMER	0.01	0.00	0.02	
	Time	0700	-	-	-	
		0900	1.04	0.74	1.47	
Grazing		1100	1.08	0.76	1.53	<0.0001
		1300	3.48	2.34	5.18	
		1500	5.73	3.68	8.92	
	Weather	Overcast	-	-	-	
		Rain	0.55	0.34	0.89	<0.0001
		Sun	0.19	0.13	0.26	
	Season	WINTER	-	-	-	
		AUTUMN	0.90	0.57	1.42	
		SPRING	1.11	0.72	1.73	<0.0001
		SUMMER	2.96	1.97	4.46	
	Time	0700	-	-	-	
		0900	1.26	0.78	2.05	
Standing		1100	1.47	0.91	2.36	0.4432
		1300	1.00	0.62	1.62	
		1500	1.21	0.76	1.92	
	Weather	Overcast	-	-	-	
		Rain	0.74	0.40	1.38	0.4354
		Sun	1.12	0.79	1.57	

## Data Availability

The original contributions presented in this study are included in the article. Further inquiries can be directed to the corresponding author.
